# Galectin-9 – ligand axis: an emerging therapeutic target for multiple myeloma

**DOI:** 10.3389/fimmu.2024.1469794

**Published:** 2024-09-25

**Authors:** Rajib K. Shil, Norhan B. B. Mohammed, Charles J. Dimitroff

**Affiliations:** ^1^ Department of Cellular and Molecular Medicine, Herbert Wertheim College of Medicine, Florida International University, Miami, FL, United States; ^2^ The Ronald O. Perelman Department of Dermatology, NYU Grossman School of Medicine, New York, NY, United States; ^3^ Department of Medical Biochemistry, Faculty of Medicine, South Valley University, Qena, Egypt

**Keywords:** galectins, multiple myeloma, galectin-9, tumor microenvironment, therapy

## Abstract

Galectin-9 (Gal-9) is a tandem-repeat galectin with diverse roles in immune homeostasis, inflammation, malignancy, and autoimmune diseases. In cancer, Gal-9 displays variable expression patterns across different tumor types. Its interactions with multiple binding partners, both intracellularly and extracellularly, influence key cellular processes, including immune cell modulation and tumor microenvironment dynamics. Notably, Gal-9 binding to cell-specific glycoconjugate ligands has been implicated in both promoting and suppressing tumor progression. Here, we provide insights into Gal-9 and its involvement in immune homeostasis and cancer biology with an emphasis on multiple myeloma (MM) pathophysiology, highlighting its complex and context-dependent dual functions as a pro- and anti-tumorigenic molecule and its potential implications for therapy in MM patients.

## 1 Introduction

**Figure 1:**
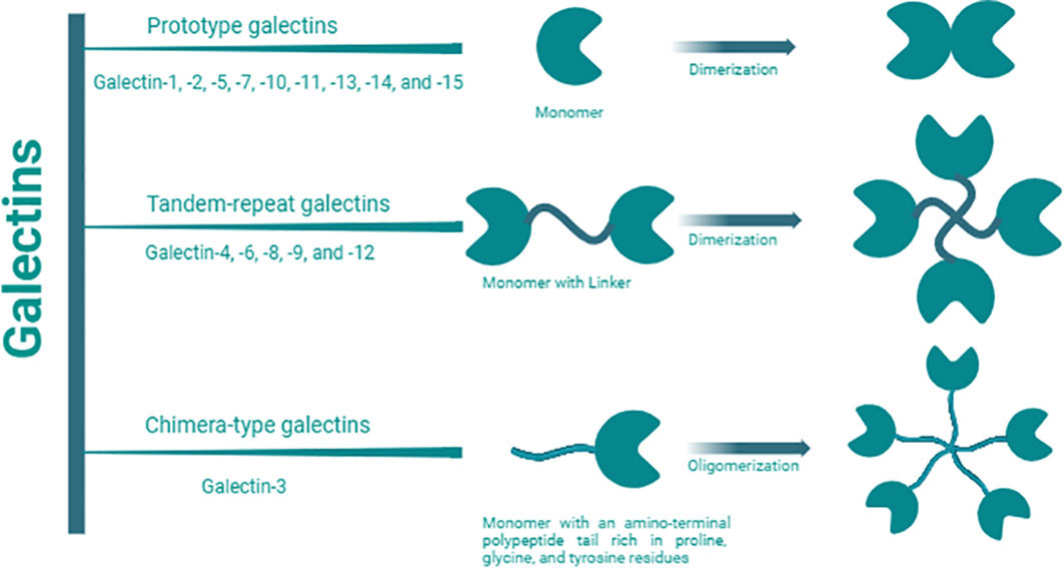
Galectin classification. Galectin members are categorized into three main groups: proto-type, chimera-type, and tandem repeat based on the number, structures, and orientation of carbohydrate recognition domains (CRDs). They specifically recognize and bind to β-galactoside on glycoconjugates (proteins, lipids, or other molecules) through these CRDs. Proto-type Gal-1, -2, -5, -7, -10, -11, -13, -14, and -15, are characterized by a single CRD which can form homodimers; chimera-type Gal-3, is composed of one CRD and an amino-terminal polypeptide tail rich in proline, glycine, and tyrosine residues for oligomer formation; and tandem-repeat Gal-4, -6, -8, -9, and -12, consist of two distinct CRDs connected by a peptide linker of variable length, ranging from 5 to over 50 amino acids.

Galectins are a family of 15 β-galactoside-binding lectins widely expressed by a wide range of mammalian cells, including immune cells (1). Galectins have been categorized into three main groups based on their molecular structures (**Figure 1**) (2). These groups include proto-type galectins (galectin (Gal)-1, -2, -5, -7, -10, -11, -13, -14, and -15), characterized by a single carbohydrate-recognition domain (CRD) that can form homodimers; chimera-type galectins (Gal-3), featuring a single CRD and an amino-terminal polypeptide tail rich in proline, glycine, and tyrosine residues for oligomer formation; and tandem-repeat galectins (Gal-4, -6, -8, -9, and -12), consisting of two CRDs connected by a peptide linker of variable length, ranging from 5 to over 50 amino acids (3, 4).

Extensive research has established galectins as important regulators of immune homeostasis (5), inflammation (6), malignancy (7–9), and autoimmune diseases (10). Considerable advancements have been achieved in understanding how galectins influence both arms of the immune response (11). In the realm of innate immunity, galectins regulate granulocyte chemotaxis, dendritic cell maturation, mast cell activation, and many other activities (6). In adaptive immunity, galectins are widely recognized for their effects on T cell function, where they differentially modulate T cell development, activation, differentiation, and effector function (6, 12, 13). However, their roles in B cell development and activation have been receiving more attention as galectins appear to have profound effects on B cell responses (11). To date, Gal-1, -3, -8, and -9 have been identified as regulators of B cell signaling (11, 14–17).

Gal-9 has been observed to exhibit both tumor-promoting and tumor-suppressing roles across various cancer types, including multiple myeloma (MM) (18, 19). This dual functionality underscores the necessity for a thorough examination of current research on Gal-9 involvement in cancer progression. In this review, we present the latest insights on this topic with particular emphasis on the role of Gal-9 in driving MM progression and its potential therapeutic implications. We urge for a more comprehensive understanding of the precise mechanisms through which Gal-9 influences MM to guide the development of future therapeutic strategies.

## 2 Galectin-9 (Gal-9)

### 2.1 Gal-9 structure, expression, and secretion

**Figure 2:**
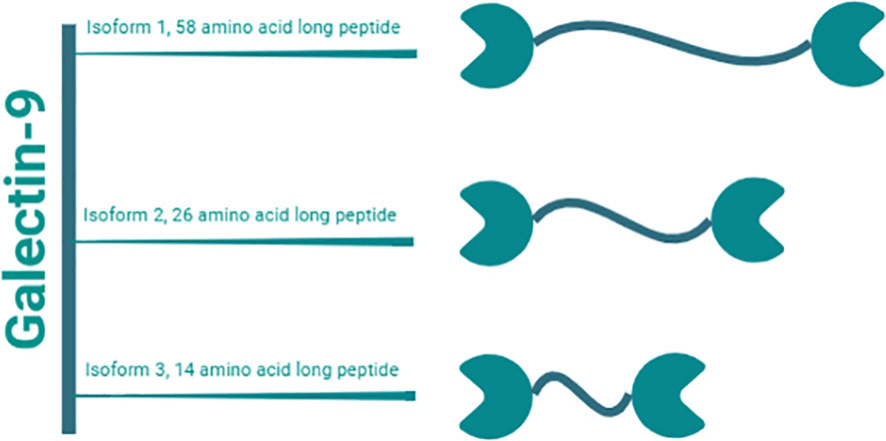
Gal-9 isoforms. Gal-9 is a tandem-repeat galectin with a molecular weight of 34 to 39 kDa. It consists of two distinct CRDs linked by a peptide of variable lengths. Humans have three natural isoforms of this lectin, which differ in the length of this interdomain peptide. These isoforms are termed i) long form Gal-9 (58 amino acids), ii) medium form Gal-9 (26 amino acids), and short form Gal-9 (14 amino acids).

Gal-9, a tandem-repeat galectin with a molecular weight of 34 to 39 kDa, has emerged as a multifaceted molecule with substantial implications in various physiological and pathological processes (20, 21). In humans, Gal-9 consists of two distinct CRDs connected by a linker peptide (**Figure 2**) (20, 22). Depending on the length of the peptide, this lectin is categorized into three types: long form (58 amino acids), medium form (26 amino acids), and short form (14 amino acids) (20). Gal-9 shows widespread distribution across multiple organs, including the liver, small intestine, thymus, kidney, spleen, lung, cardiac, and skeletal muscles, with minimal detectability in reticulocytes and brain tissues (23). Gal-9 is primarily expressed by various immune cells including T cells, B cells, macrophages, and mast cells (24, 25). Moreover, under both normal and pathophysiological conditions, Gal-9 can also be detected in endothelial cells, fibroblasts, and astrocytes (14, 17, 26–28).

Gal-9 can be found either extracellularly or intracellularly within the nucleus (29–31) as well as in the cytoplasm (21, 29, 32). While cell surface Gal-9 plays various roles in different cellular processes such as cell aggregation and apoptosis, its cytoplasmic functions are still not fully understood (31, 32). Gal-9 secretion into the extracellular space does not follow the classical secretory pathway due to the absence of the signal secretion peptide essential for its transport into the endoplasmic reticulum (33, 34). The exact mechanism by which Gal-9 is secreted is not fully understood; however, it is proposed to be released from the cell via non-classical secretory pathways involving matrix-metalloproteinases and protein kinase C (PKC) (20). These pathways may include translocation directly across the plasma membrane, release via exosome packaging, or export via lysosomes, endosomes, and microvesicles (35–37).

### 2.2 Gal-9 and its glycoconjugate ligands

Gal-9 binds to β-galactoside-bearing glycoconjugates on the cell surface through its CRDs, influencing diverse cellular functions and signaling pathways (38–43). The N- and C-terminal CRDs of Gal-9 exhibit only 39% amino acid similarity and interact with distinct sets of carbohydrate ligands (28, 44). The varying specificity and affinity of both the N- and C-terminal CRDs for specific glycoconjugates directly influence Gal-9 affinity toward its binding partners (45, 46). For instance, the N-terminal CRD is suggested to play a predominant role in activating dendritic cell function, while the C-terminal CRD is mainly involved in triggering T-cell death (47). Moreover, variations in the length of the linker domain between the two CRDs affect their rotational flexibility, facilitating the formation of higher-order multimers and increasing Gal-9 valency (48, 49). Earl et al. experimentally demonstrated that incorporating the linker region from Gal-9 into constructs of Gal-1 dimer alters their potency in killing T cells, while the glycan-binding specificity of the CRD remains unchanged compared to the wild-type galectin (49). The broad range of functions carried out by Gal-9 stems from its ability to interact with multiple partners (**Table 1**). These binding partners range from intracellular cytosolic/nuclear proteins to canonical membrane glycoproteins uniquely expressed in a cell- or tissue-specific manner (30, 43, 62). Moreover, glycosylation of these Gal-9 glycoprotein ligands is often contextualized in relation to the coordinated expression of glycosyltransferases associated with cell differentiation or a malignant or metastatic cell transition (15, 72).

**Table 1:** Gal-9 binding partners.

Binding partner	Biological function	Reference
Lipid rafts	Induction of osteoblast differentiation	(50, 51)
Forssman glycosphingolipid (FGL)	Maintenance of epithelial polarity	(52)
Latent membrane protein 1	Not Determined	(53)
Poly-β-galactosyl epitope (Galβ1–3)n	*Promote Leishmania major* and host interaction	(54)
T cell immunoglobulin mucin-3 (Tim-3)	Maturation, promotion, and cytokine secretion of dendritic cells, monocytes Exhaustion or apoptosis of T cells	(55, 56) (57, 58)
Thrombin	Cleavage of Gal-9 and decrease of eosinophil attraction	(59)
Gal-3	Hetero/multimerization and increased valency	(60)
Gal-8	Hetero/multimerization and increased valency	(60)
Gal-9	Homo/multimerization and increased valency	(60)
CD40	Suppressing T cell proliferation and inducing cell death	(61)
CD44	Suppression of CD44-hyaluronic acid binding Induces differentiation and maintenance of adaptive regulatory T cells. Osteoblast differentiation through the CD44/Smad signaling pathway	(62) (63) (50)
CD45	Regulating B cell activation	(15)
CD206	Driving angiogenesis and producing chemokines to support tumor growth by macrophages	(64)
Immunoglobulin E (IgE)	Prevention of mast cell degranulation and suppression of the allergic response	(39)
Immunoglobulin M (IgM)	Regulating B cell signaling	(16)
Protein disulfide isomerase (PDI)	Regulating T cell migration	(40)
Programmed cell death protein 1 (PD-1)	Attenuate Gal-9/TIM-3-induced T cell apoptosis	(65)
Dectin-1	Tolerogenic macrophage programming and adaptive immune suppression	(66)
Vascular cell adhesion molecule-1 (VCAM-1)	Suppression of VLA-4/VCAM-1 binding	(67)
V-domain Ig-containing suppressor of T cell activation (VISTA)	Apoptosis of cytotoxic T cells	(68)
Glucose transporter 2 (GLUT- 2)	Maintenance of blood glucose homeostasis and suppression of type 2 diabetes onset	(41)
Glucagon receptor (Gcgr)	Promoting hyperglycemia	(38)
4-1BB (CD137)	Facilitate signaling and functional activity in T cells, dendritic cells, and natural killer cells	(69)
NF-IL6 (C/EBP β)	Inflammatory cytokine production in monocytes	(30)
DR3	Promoting Treg function that dampen inflammatory disease	(70)
Toll-like receptor-4 (TLR-4)	Alleviating brain injury and promoting neuronal restoration by microglial activation	(71)

### 2.3 Gal-9 role in immune homeostasis

Galectins are now well-recognized for playing critical roles in both innate and adaptive immune responses (73). Gal-9 initiates innate immunity by promoting the maturation of dendritic cells, as evidenced by the increased expression of Th1 cytokines and co-stimulatory molecules, including HLA-DR, CD83, CD80, CD54, and CD40. Subsequently, these matured dendritic cells migrate to lymph nodes, where they trigger the activation of T cells (55). In addition, Gal-9 acts as a chemoattractant for eosinophils, modulates signal-dependent chemotaxis of neutrophils, and enhances phagocytosis (27, 74). In monocytes, intracellular Gal-9 induces the transcription of the proinflammatory cytokines IL-1α, IL-1β, and IFN-γ (30). Conversely, Gal-9 also promotes the expansion of immunosuppressive macrophages (75).

In adaptive T cell-mediated immune responses, Gal-9 plays a crucial role in regulating T cell development and homeostasis (76). It has diverse immunomodulatory effects depending on the concentration, receptors, and skewing signals available for the interactions (77). At high concentrations, Gal-9 induces apoptosis of activated T cells (CD8^+^ and CD4^+^), while it increases cytokine production by activated T cells at low concentrations (77, 78). Additionally, Gal-9 facilitates the differentiation of naïve T cells into regulatory T cells (Tregs) by amplifying Foxp3 expression, nevertheless inhibiting the production of Th17 cells, thus participating in Th17/Treg immune regulatory functions (79).

Similarly, galectins are also crucial in signal transduction and the regulation of B cell development, differentiation, activation, and antibody production (14). In particular, Gal-9 can compromise B cell activation transmitted via the B cell receptor (15, 16). In humans, the interaction between Gal-9 and appropriated glycosylated CD45 on human naïve/memory B cells inhibits calcium signaling through a Lyn-CD22-SHP-1 pathway, ultimately reducing B cell activation (15). Additionally, both recombinant and mesenchymal stem cell-derived Gal-9 can attenuate B cell proliferation and the formation of antibody-secreting cells in a dose-dependent fashion (80). These findings are further solidified by studies from Hu et al. that show human cord blood-derived stem cells directly modulate activated B cells through a Gal-9-mediated mechanism, resulting in significant suppression of B cell proliferation and noticeable phenotypic changes (81). In addition, Gal-9 can bridge human circulating and naive B cells to vascular endothelial cells, coordinating their pace of transendothelial migration (17). Furthermore, these interactions induce a global transcriptional response in gene families associated with the regulation of naive B cell signaling and membrane/cytoskeletal dynamics (17). Among the key immunoregulators elevated by Gal-9 binding is the signaling lymphocytic activation molecule family member 7 (SLAMF7), while its cytosolic adapter EAT-2 necessary for cell activation is unaffected (17). Moreover, Gal-9 can encourage the survival traits of human naïve and circulating B cells by inducing the phosphorylation of the pro-survival factor, pERK (17).

## 3 Gal-9 involvement in malignancy

Central to the development of effective therapies for eliminating cancer is the exploration of malignancy-associated molecules and their role in driving tumor development, metastasis, and relapse (82). The patterns of Gal-9 expression and its roles in various types of cancer have emerged as a compelling area of research, aiming to deepen our comprehension of mechanisms underlying cancer initiation and progression and identify novel therapeutic targets (83, 84). Gal-9 exhibits substantial variability in its expression levels across different types of cancer (83). Compared to healthy tissues, Gal-9 is upregulated in pancreatic carcinoma, breast cancer, glioblastoma multiforme, cervical carcinoma, chronic lymphocytic leukemia, acute myeloid leukemia (AML), and cutaneous T cell lymphoma (CTCL) (85–91). In B-cell acute lymphoblastic leukemia (B-ALL) patients, while serum Gal-9 levels remain unchanged compared to healthy controls, Lee et al. demonstrated that adipocyte-secreted factors in obese patients upregulate Gal-9 surface expression on B-ALL cells (91, 92). Conversely, Gal-9 expression is notably downregulated in esophageal carcinoma, renal cell carcinoma, hepatocellular carcinoma, colon cancer, gastric cancer, prostate cancer, lung cancer, melanoma, and adrenal carcinoma compared to healthy counterparts (31, 93–97).

Additionally, there has been a growing interest in exploring the prognostic value of Gal-9 expression levels in patients with cancer (83). Many studies suggest an inverse relationship between Gal-9 expression levels and cancer progression for many solid tumors, including esophageal carcinoma, gastric cancer, hepatocellular carcinoma, colorectal cancer, lung cancer, breast cancer, and melanoma (93, 98–105). In these instances, administering exogenous Gal-9 can effectively impede the growth of these tumors, representing a promising anti-cancer treatment (83). However, contradictory findings exist where a few studies associate high tissue and plasma Gal-9 with poor survival and clinical outcomes (90, 106). Indeed, Gal-9 and its binding partner, T cell immunoglobulin mucin-3 (Tim-3), have been implicated in negatively regulating the cellular immune response by inducing T cell apoptosis and exhaustion, fostering an immunosuppressive tumor microenvironment (TME) (18, 43). Interestingly, in non-small cell lung carcinoma, high Gal-9 expression on cancer cells is associated with longer overall survival, whereas elevated Gal-9 levels on tumor-infiltrating lymphocytes predict shorter recurrence-free survival (101). Hence, these opposing effects are likely reflected by context-dependent roles of Gal-9 in cancer, influenced by its localization within the TME, by cancer-specific responses, by tumor heterogeneity, by variability in the expression profiles of Gal-9 glycoconjugates ligands, and by diversity of experimental results driven by differing methods of analysis and inadequate clinical sample sizes (107). These variabilities of Gal-9 expression not only influence tumor development and progression, but also patient prognosis (**Table 2**).

**Table 2:** Gal-9 expression in cancer and its prognostic value.

Gal-9 as Promoter of Tumorigenicity		
Cancer Type	Putative mechanism	Reference
Pancreatic carcinoma	Immunosuppression via macrophage reprogramming	(66, 87, 108)
Cervical carcinoma	Tumor immune evasion	(86, 109)
Glioma	Tumor immune evasion	(88, 110)
Leukemia	Tumor immune evasion	(91, 111, 112)
Lymphoma	Decreases CD8+ T cell infiltration	(89)
Gal-9 as a Tumor Suppressor		
Cancer Type	Putative mechanism	Reference
Esophageal carcinoma	Promotes tumor cell apoptosis	(97, 102, 113)
Colorectal cancer	Promotes tumor cell apoptosis, immune surveillance, autophagy, and suppresses proliferation	(93, 114–116)
Gastric cancer	Promotes tumor cell apoptosis	(100, 117)
Lung cancer	Inhibits tumor cell adhesion and invasion while activating NK cells	(67, 101, 118)
Gal-9 with Both Tumorigenic and Tumor Suppression Activity		
Cancer Type	Putative mechanism	Reference
Breast cancer	Pro-Tumor – Inhibits immune surveillance Anti-Tumor – Decrease metastasis	(85, 103, 119)
Multiple myeloma	Pro-Tumor – Promotes tumor immune evasion Anti-Tumor – Suppresses tumor cell proliferation and induces tumor cell apoptosis	(18, 19, 120)
Hepatocellular carcinoma	Pro-Tumor – Promotes tumor growth and metastasis Anti-Tumor – Induces tumor cell apoptosis	(95, 98, 121–124)
Melanoma	Pro-Tumor – Promotes M2 macrophages-mediated angiogenesis Anti-Tumor – Suppresses tumor cell invasion and metastasis and induces tumor cell apoptosis	(31, 64, 67, 125, 126)

### 3.1 Gal-9 and multiple myeloma (MM)

#### 3.1.1 Characteristics and diagnosis of MM

MM is a clonal B cell malignancy, accounting for approximately 1% of all cancers and 10% of hematologic malignancies (127). It is characterized by abnormal accumulation of ≥10% malignant clonal plasma cells in the bone marrow (128–132). MM is commonly diagnosed in elderly individuals, with a median age at diagnosis around 66 years (133). There is a clear sex disparity in MM incidence across all ages and racial/ethnic groups, with males showing higher incidence and mortality rates compared to females. However, the underlying mechanisms and the impact of sex on patient outcomes remain poorly understood and underexplored (134, 135). Data from a recent study suggest that individuals with MM have a median survival rate of about 7.5 years (136). Most individuals with MM exhibit the production of a monoclonal immunoglobulin protein, also termed M-protein, generated by aberrant clonal plasma cells. Nonetheless, in 15–20% of cases, MM cells exclusively release monoclonal free light chains, and in fewer than 3% of patients, these cells do not secrete any monoclonal protein (133, 137).

The malignant clonal plasma cells in MM cause typical end-organ damage, such as hypercalcemia, renal failure, anemia, and lytic bone lesions referred to as “CRAB” symptoms (138–140). The presence of one or more of the CRAB symptoms sets MM apart from other plasma cell disorders, such as smoldering multiple myeloma (SMM) and monoclonal gammopathy of undetermined significance (MGUS) (138). SMM is identified by 10-60% bone marrow plasma cell proliferation and/or elevated M-protein ≥3 g/dL, without CRAB symptoms (141), while MGUS is characterized by a clonal bone marrow plasma cells <10% and a serum M-protein level <3 g/dL in asymptomatic patients (142). Both SMM and MGUS are recognized as precursors to MM, with an annual progression risk of 10% and 1%, respectively (143).

The surface antigens CD138, CD38, CD45, CD19, CD56, CD117, CD20, CD28, CD27, and CD81 are the most widely used markers for characterizing normal and malignant plasma cells (144). BM-derived myeloma cells are typically defined by their high expression of CD38, CD138, and CD56, low expression of CD45, and absence of CD19 (145). In addition to its crucial role in establishing MM diagnosis, numerous studies have reported the prognostic and therapeutic significance of plasma cell immunophenotypic features in MM patients (146). For instance, the upregulation of CD19 and its regulatory partner CD81 are linked to poorly differentiated plasma cell clones associated with poor outcomes in MM patients (147). Similarly, the expression of CD45 on clonal plasma cells is associated with an aggressive phenotype of MM (148). B cell maturation antigen (BCMA/CD269) is another surface marker that is primarily expressed by plasma cells and mature B lymphocytes (149, 150), and is upregulated on malignant plasma cells in MM (151, 152). BCMA is a target antigen for the FDA approved chimeric antigen receptor-T (CAR-T) cell therapies for MM (153).

#### 3.1.2 Mechanistic roles of Gal-9 across diverse malignancies

In breast cancer, Gal-9 overexpression enhances cancer cell adhesion and invasion through Focal Adhesion Kinase activation, which upregulates the pro-invasive protein S100A4, and facilitates immune evasion via the Tim-3-Gal-9 pathway by transferring Gal-9 to the cell surface (85, 154–156). In contrast, recombinant protease-resistant Gal-9 (rGal-9) exerts anti-proliferative effects in gastric cancer by inducing apoptosis, reducing the phosphorylation of VEGFR-3 and IGF-1R, and altering miRNA expression (100). In colorectal cancer, Gal-9 promotes extensive intra-tumoral NK cell infiltration through Rho/ROCK1 signaling, correlating with improved prognosis (93, 157, 158). Similarly, in mice with lung cancer, Gal-9 induces macrophage differentiation into plasmacytoid dendritic cell-like macrophages, potentially enhancing NK cell activation, and extending longevity (118). Gal-9 also inhibits tumor growth in hepatocellular carcinoma by inducing apoptosis via MicroRNAs mediated miR-1246-DYRK1A-caspase-9 axis (121), and suppresses esophageal squamous cell carcinoma proliferation both *in vitro* and *in vivo* through Jun NH2-terminal kinase (JNK) and p38 MAPK pathways (113). However, Gal-9 may support tumor immunosuppression in glioblastoma by promoting M2 tumor-associated macrophage activity (88). In contrast, in pancreatic ductal adenocarcinoma, Gal-9’s binding to Dectin-1-expressing macrophages fosters immunosuppression and tumor progression, though exogenous rGal-9 can induce apoptosis by promoting cytochrome release and altering miRNAs expression in this context (66, 159). In cervical cancer, the Tim-3-Gal-9 pathway facilitates immune escape by promoting regulatory T cell activity, with epigenetic regulation through the H3K9me3-specific histone methyltransferase (SUV39H1) - DNA methyltransferase 3 alpha (DNMT3A) axis (86, 109). While serum Gal-9 levels are elevated in patients with advanced cutaneous T-cell lymphoma, the exogenous administration of rGal-9 induces apoptosis by Tim-3 independent activation of caspase-3 and caspase-9, and suppresses *in vivo* tumor growth (89). Finally, in AML, the Tim-3-Gal-9 autocrine loop promotes leukemic stem cell self-renewal and disease progression through the NF-κB, β-catenin, and PKC/mTOR pathways (91, 111). These findings underscore the complex and context-dependent roles of Gal-9 in cancer biology, highlighting its potential therapeutic importance in MM.

#### 3.1.3 The BM microenvironment in MM

The BM consists of a heterogeneous population of cells, including hematopoietic stem cells and their myeloid and lymphoid progeny, along with endothelial cells and cells derived from mesenchymal stromal cells, such as adipocytes, chondrocytes and osteoblasts, all of which are embedded in the extracellular matrix (ECM) (160). In MM, the crosstalk between MM cells and the surrounding BM microenvironment activates intracellular signaling pathways that impact tumor behavior, including tumor growth, progression, angiogenesis, immune evasion, and drug resistance (161, 162). This crosstalk is facilitated by direct cell-cell contact through surface adhesion molecules, secreted soluble cytokines and growth factors, and exosome-mediated intercellular communication (163).

Different molecular complexes on MM cells interact with ECM proteins, such as syndecan-1 (CD138) with collagen type 1 (164) and very late antigen-4 (VLA-4) with fibronectin (165), or with their binding partners on various non-tumor cells, such as VLA-4 with vascular cell adhesion molecule-1 (VCAM-1) and lymphocyte function-associated antigen-1 (LFA-1) with intercellular adhesion molecule-1 (ICAM-1) (166). These interactions activate various downstream signaling pathways associated with tumor progression and poor outcomes in MM patients such as NF-κB, ERK, and phosphoinositide 3-kinases (PI3K)/Akt pathways (163). Cytokines and growth factors, synthesized and released by bone marrow stromal cells (BMSCs) or MM cells, are also implicated in triggering the major signaling pathways that promote the survival and dissemination of MM cells and mediate drug resistance (166). Key MM-associated cytokines include interleukin-6 (IL-6), IL-10, IL-17a, insulin-like growth factor-1 (IGF1), vascular endothelial growth factor (VEGF), fibroblast growth factor (FGF), transforming growth factor-β (TGF-β), and B cell-activating factor (BAFF) (167–169). Cytokines are also involved in disrupting normal bone remodeling in MM, leading to increased bone resorption and formation of osteolytic lesions, which are present in approximately 80% of MM patients (170). Both BMSCs and MM cells contribute to the pathogenesis of myeloma osteolytic lesions through secretion of osteoclast activating factors, such as receptor activator of nuclear factor-κB (RANKL), tumor necrosis factor-α (TNF-α), macrophage inflammatory protein-1α (MIP-1α), macrophage colony-stimulating factor (MCSF), IL-3, and IL-6 (171) as well as osteoblast inhibitory factors such as the Wnt antagonists Dickkopf-1 (DKK1), secreted frizzled-related protein-2 (sFRP-2), Runt-related transcription factor 2 (Runx2), and TGF-β (172).

Characteristically, MM cells foster an immunosuppressive bone marrow environment not only by inhibiting antitumor effector cells and disrupting antigen presentation, but also by encouraging the expansion of regulatory immune cells (173), such as myeloid-derived suppressor cells (174, 175), Tregs (176, 177), and regulatory B cells (178, 179). The myeloma microenvironment also shows a significant increase in Th17 T cells, probably due to the myeloma cell-derived IL-6 and TGF-β production (180). Increased IL-17 production by Th17 T cells has been demonstrated to suppress cytotoxic T cell activity, promote myeloma cell growth *in vitro* and *in vivo* through IL-17 receptors (IL17R), and play a crucial role in MM-associated bone disease (180, 181). Moreover, dendritic cells (DCs) exhibit functional impairment in MM patients, characterized by the inability to upregulate CD80 expression in response to stimulation by human CD40LT and IL-2, possibly due to their inhibition by the elevated levels of TGFβ1 and IL-10 in the myeloma microenvironment (182, 183). Importantly, studies have demonstrated an upregulated programmed death ligand-1 (PD-L1) on malignant plasma cells is reported to increase as MGUS progresses to MM (184), combined with elevated expression of its receptor programmed death 1 (PD-1) on several MM-associated immune cell subsets, including T cells, B cells, NK cells, and DCs (185). The interaction between PD-L1 and PD-1 represents a major contributor to the immunosuppressive characteristics observed in the MM microenvironment and is shown to be counteracted by PD-L1 or PD-1 blockade (186, 187).

#### 3.1.4 Dual role of Gal-9 in MM

**Figure 3:**
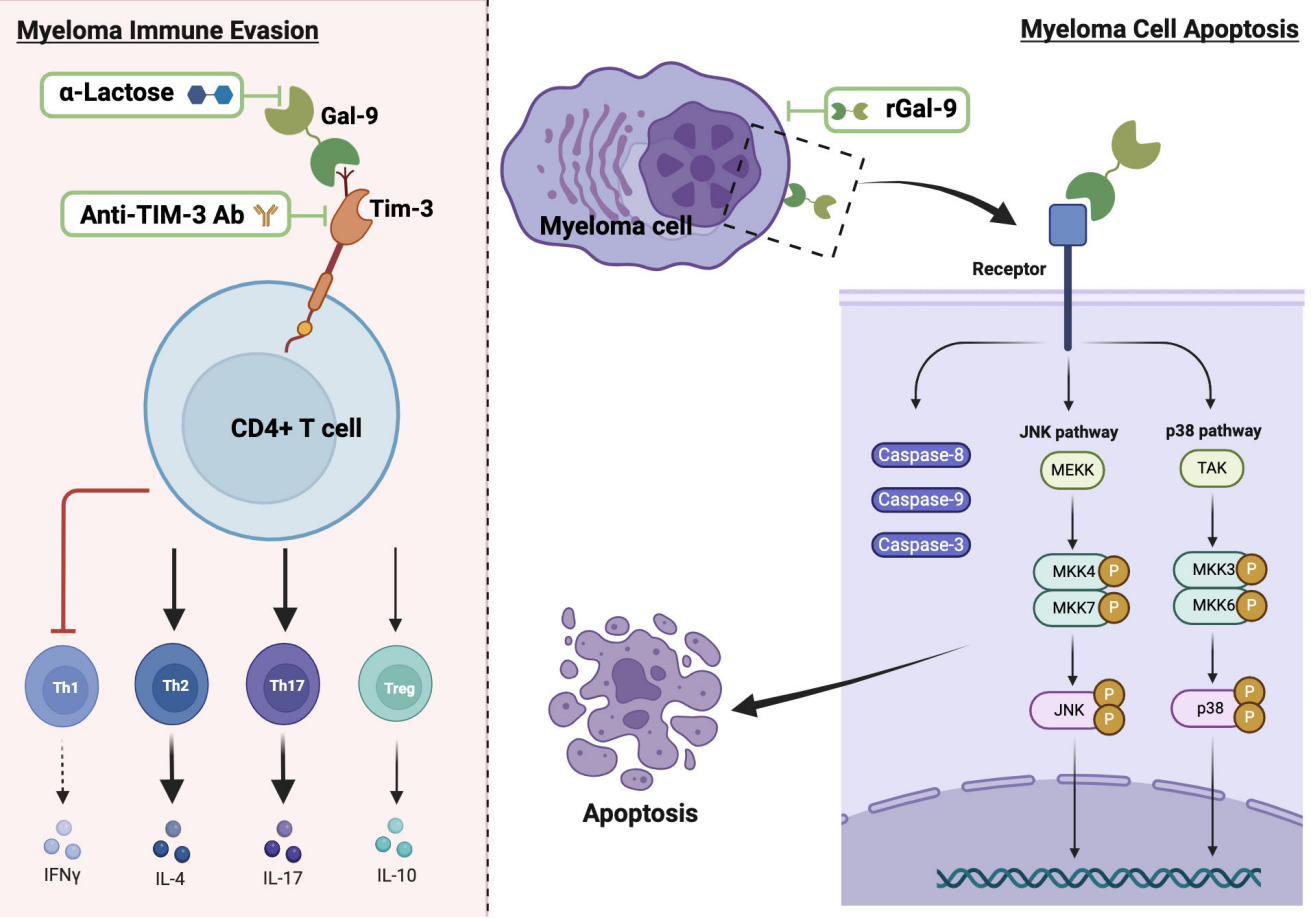
The dual role of Gal-9 in MM and potential therapeutic strategies. The left panel depicts the pro-tumorigenic effect of Gal-9, showing its binding to Tim-3 on CD4+ T cells, which leads to disrupted T cell subset balance by inhibiting Th1 and enhancing the immune response of Th2 and Th17 cells. The right panel illustrates the anti-tumorigenic effect of Gal-9, demonstrating its binding to myeloma cells and subsequent activation of apoptotic pathways through caspases and JNK/p38 MAPK signaling. Thick black arrows indicate activation and bar-ended red lines represent inhibition. Potential therapeutic strategies are shown within green frames.

Data from MM studies on the role of Gal-9 are sparse and controversial, with evidence documenting both pro-tumorigenic and anti-tumorigenic activities (**Figure 3**) (18, 19). Investigations conducted by Lee et al. on BM aspirate samples obtained from 109 newly diagnosed MM patients indicate that Gal-9 expression is an independent predictor of poor survival in patients who exhibit elevated PD-L1 levels (107). In a recent study performed on blood samples from 60 newly diagnosed MM patients and 40 healthy controls, Zhang et al. have revealed upregulated expression levels of Tim-3 on CD4+ T cell surfaces, elevated Gal-9 mRNA in peripheral blood mononuclear cells, and increased serum levels of Gal-9 in MM patients compared to healthy donors (18). The researchers have demonstrated that Tim-3, upon binding with Gal-9, disrupts the balance between CD4+ T cell subsets (Th1, Th2, Th17, and Treg cells) and impacts secretion of their cytokines, leading to inhibition of the cytotoxic function of Th1 cells and promotion of Th2 and Th17 cells involvement in the immune escape of MM (18).

On the other hand, earlier studies led by Kobayashi et al. report that rGal-9 effectively suppresses the proliferation of various human B-lymphoblast myeloma cell lines (HMCL), namely IM9, AMO-1, KMS-12-BM, NCI-H929, and RPMI8226, and its effectiveness is directly associated with the binding affinity of Gal-9 to each HMCL (19). Their data also demonstrate that Gal-9 exerts a dose-dependent pro-apoptotic effect on primary myeloma cells derived from 10 MM patients, including cells from treatment-resistant patients with poor prognosis and chromosomal abnormalities (19). Mechanistically, the authors suggest that Gal-9 induces apoptosis through the activation of caspase-8, -9, and -3, associated with the activation of JNK and p38 MAPK signaling pathways, while inhibiting JNK or p38 MAPK pathways result in attenuated the anti-proliferative effect of Gal-9, highlighting the crucial role of these pathways in mediating the anti-myeloma cell effect of Gal-9 (19). In contrast, data from another study reveal that Gal-9 induces T cell apoptosis with no effect on MM cells (120). The dual role of Gal-9 extends beyond MM; for instance, in hepatocellular carcinoma, its downregulation in hepatocytes promotes tumor growth and metastasis, while its overexpression in Kupffer cells and endothelial cells suppresses the anti-tumor immune response by inducing T cell apoptosis or senescence (98, 121–123). This duality may be due to the loss of Gal-9 expression during tumor progression after its initial upregulation helps establish a tumorigenic environment (122). In malignant melanoma, high Gal-9 expression suppresses the invasion and metastasis by blocking adhesion to endothelium and ECM (67). However, in metastatic malignant melanoma patients, Gal-9 binding to CD206 expressing M2 macrophages promotes tumor growth by increased angiogenesis and chemokine production (64). Further research is required to explore how Gal-9 contributes to each of the cancer hallmarks in MM patients, considering its cellular and subcellular localizations and its interacting partners within the TME. Additionally, the potential significance of individual Gal-9 splice variants in regulating MM progression needs to be elucidated.

#### 3.1.5 Clinical implications of Gal-9 in MM

The standard treatment for MM involves a variety of drug combinations aiming to enhance patient survival and quality of life (188, 189). These include alkylating agents (melphalan, cyclophosphamide), corticosteroids (dexamethasone, prednisone), anthracyclines (doxorubicin and liposomal doxorubicin), proteasome inhibitors (PIs; bortezomib, carfilzomib, ixazomib), immunomodulatory drugs (IMiDs; thalidomide, lenalidomide, pomalidomide), monoclonal antibodies (mAbs; Daratumumab, isatuximab, elotuzumab), CAR-T cell therapies, nuclear export inhibitors, histone deacetylase inhibitors (iHDACs), and autologous stem cell transplantation (ASCT) (190–192). The selection of therapy throughout the disease course is influenced by factors such as age, performance status, comorbidities, eligibility for stem cell transplantation, and the risk stratification of MM patients (189). The strategic use of these treatment modalities has significantly enhanced the overall survival rates in MM patients (193–195). However, challenges, such as drug intolerability, drug resistance, and disease-relapse remain prevalent, necessitating the ongoing development of novel therapeutic strategies (196).

Prior studies have underscored the critical role of Gal-9 and its binding partner Tim-3 on CD4+ T cell surfaces in the development of treatment resistance in both hematologic and solid malignancies (197). In AML, patients who failed to respond to chemotherapy exhibited elevated expression levels of Gal-9 and Tim-3, suggesting that targeting the Gal-9/Tim-3 axis could potentially enhance the effectiveness of induction chemotherapy and improve remission rates in AML patients (198). Furthermore, a growing body of evidence from both preclinical studies and clinical trials indicates that combination therapy targeting Gal-9/Tim-3 alongside anti-PD-1/PD-L1 treatment produces superior outcomes in cancer patients compared to either approach alone (83).

These promising results in various cancer types spark interest in exploring the potential of Gal-9/Tim-3 targeting in MM. While comprehensive studies specifically examining the combination of Gal-9 inhibitors with standard anti-MM therapies are currently lacking, the substantial evidence of Gal-9 contribution to an immunosuppressive TME in MM strongly suggests the potential for synergistic therapeutic effects (18). For instance, blocking Gal-9/TIM-3 interactions could potentially enhance the efficacy of FDA-approved BCMA-targeted CAR-T cell therapies by prolonging their survival and activity within the TME, resulting in a more effective elimination of MM cells (199). This approach could be particularly beneficial in MM, where maintaining CAR-T cell function in the hostile TME remains a significant challenge (200). Likewise, combining Gal-9 inhibition with the lenalidomide, a standard anti-MM therapy that enhances the cytotoxic activities of T-cells and NK-cells against tumor cells, could potentially augment the immune response against MM (201). However, this combination strategy warrants further investigation.

In this regard, blocking Tim-3 using mAbs or small molecules has shown promise in preclinical studies (202–204). Similarly, α-Lactose and its derivatives have been used for targeting Gal-9 (205, 206). However, concerns about their non-selective inhibition of galectins and widespread presence in food and pharmaceutical preparations emphasize the need for alternative Gal-9 inhibitors (206). Recently, researchers have developed novel Gal-9-neutralizing antibodies that protect T cells from Gal-9-induced cell death and enhance T cell-mediated tumor cell killing (207).

On the other hand, considering the pro-apoptotic effect of rGal-9 on MM cells, there is a possibility of targeting MM through the development of stable rGal-9 formulations for delivery to myeloma cells (19). Indeed, Kobayashi et al. have demonstrated significant efficacy and relative safety of Gal-9 as a tumor growth inhibitor in MM xenograft models, suggesting its potential as a therapeutic approach for MM (19). Notably, exogenous administration of Gal-9 has been shown to effectively induce tumor cell apoptosis in other types of cancer, such as esophageal cancer (113), hepatocellular carcinoma (121), gastric cancer (100), chronic myeloid leukemia (208), and CTCL (89). Furthermore, rGal-9 administration has been shown to inhibit tumor progression by impeding the *in vivo* metastasis of highly metastatic melanoma and colon cancer cells (67). Prior studies have also demonstrated that exogenous Gal-9 treatment significantly increases the expression of the B cell immunoregulatory factor SLAMF7, which represents a promising immunotherapeutic target for MM (17). Accordingly, combining rGal-9 with anti-SLAMF7 therapy holds the potential for synergistically enhancing the targeting of MM cells (209, 210). However, alterations in the cell glycome during the transition from normal plasma cells to myeloma cells likely impact Gal-9 binding affinity and modify intracellular signaling activation, highlighting the necessity for thorough investigation of this hypothesis (211, 212). Additionally, combining rGal-9 with proteasome inhibitors, which disrupts protein degradation in myeloma cells, could yield synergistic effects in inducing apoptosis through the simultaneous targeting of complementary cell death pathways (213). Further research is needed to fully elucidate the potential of Gal-9-based therapy, identify patients who would benefit most, and develop novel combination strategies for MM patients to overcome current treatment challenges and improve patient outcomes.

## 4 Conclusions

Gal-9 is a ubiquitous molecule predominately expressed by immune and stromal cells of tissues in the immune system. It interacts with a diverse array of intracellular, cell membrane and extracellular ligands, influencing numerous cellular processes that govern development/regulation of immune responses and development/progression of a malignancy. A growing body of evidence, in fact, indicates that the Gal-9 - ligand axis considerably contributes to the pathophysiology of many cancer types. In MM, the Gal-9 ligand-binding activity causes both tumor-promoting and tumor-suppressive properties depending on the appropriately glycosylated ligands on discrete cell types in the TME. Further research is warranted to elucidate the precise mechanisms underlying the dual role of Gal-9 in MM, paving the way for targeting Gal-9 in combination with currently available anti-MM therapies to enhance the treatment efficacy. Translating these findings into clinical applications holds promise for improving patient care and outcomes of this challenging hematologic malignancy.
